# Sanguinarine protects against osteoarthritis by suppressing the expression of catabolic proteases

**DOI:** 10.18632/oncotarget.17036

**Published:** 2017-04-11

**Authors:** Yan Ma, Xuewu Sun, Kangmao Huang, Shuying Shen, Xianfeng Lin, Ziang Xie, Jiying Wang, Shunwu Fan, Jianjun Ma, Xing Zhao

**Affiliations:** ^1^ Department of Orthopaedic Surgery, Sir Run Run Shaw Hospital, Medical College of Zhejiang University, Hangzhou 310016, China; ^2^ Key Laboratory of Biotherapy of Zhejiang Province, Hangzhou 310016, China

**Keywords:** sanguinarine, chondrocyte, osteoarthritis, catabolic proteases

## Abstract

Inflammatory cytokines play critical roles in the pathogenesis of osteoarthritis. Recent studies have demonstrated that natural active substances can serve as alternative therapeutic agents for the prevention and treatment of osteoarthritis. Sanguinarine, an alkaloid isolated from the roots of *Sanguinaria canadensis*, is known to have anti-inflammatory properties. The aim of the present study was to investigate the therapeutic effect of Sanguinarine against osteoarthritis. Sanguinarine inhibited interleukin-1β-induced expression of matrix metalloproteinase 1, 3, and 13, and A disintegrin and metalloproteinase with thrombospondin motifs-5 in chondrocytes, which involved the nuclear factor-κB and c-Jun N-terminal kinase signalling pathways. Furthermore, the study of interleukin-1β-induced cartilage matrix degradation in an anterior cruciate ligament transection-induced osteoarthritis model revealed that Sanguinarine ameliorated osteoarthritis by inhibiting the expression of matrix metalloproteinase 1, 3, and 13, and A disintegrin and metalloproteinase with thrombospondin motifs-5. In conclusion, we demonstrated for the first time that Sanguinarine suppressed the expression of matrix metalloproteinase 1, 3, and 13, and A disintegrin and metalloproteinase with thrombospondin motifs-5 *in vitro*, *ex vivo*, and *in vivo*, indicating its potential usefulness in treating osteoarthritis.

## INTRODUCTION

Osteoarthritis (OA) is a degenerative joint disease characterised by cartilage damage, subchondral bone remodelling and associated inflammation [1]. It is the most common form of arthritis and is accompanied by joint pain and stiffness, which can affect work and normal daily activities. Oral or topical nonsteroidal anti-inflammatory drugs or tramadol can relieve OA symptoms [2]. However, joint replacement surgery is often the only effective treatment for terminal OA. Considering that few pharmacological therapies can delay OA development, new strategies for the treatment of OA are urgently needed.

Several studies have suggested that OA development is related to age, obesity, inflammation, trauma, and hereditary factors [3, 4], but the fundamental causes of OA remain unclear. Inflammatory cytokines such as interleukin (IL)-1β and tumour necrosis factor (TNF)-α have been reported to be involved in the development of OA [5]. Therefore, inhibition of inflammation may be effective in treating OA. However, newer biological therapies such as anti-TNF-α therapy are very expensive and could have numerous side effects [6]. Natural compounds have recently attracted increasing attention owing to their various bioactivities in human diseases. Several natural compounds including schisantherin A, thymoquinone, taraxasterol, sesamin, and astragalin have shown anti-OA properties [7–11], and our reach group has had a long-standing interest in the application of natural compounds [12–14].

Sanguinarine (SA), an alkaloid found in the roots of *Sanguinaria canadensis*, is approved by the U.S. Food and Drug Administration (FDA) [15]. It presents a wide range of biological activities including anti-inflammatory, antitumour, antimicrobial, anti-platelet, and anti-hypertensive effects, as well as inhibition of osteoclast formation [16–19]. *Sanguinaria*-containing toothpaste and oral rinse products have been used to reduce plaque and gingival inflammation [19].

In the present study, considering its widespread use and potential for suppressing inflammation, the therapeutic effect of SA against OA was investigated *in vitro*, *ex vivo*, and *in vivo* to determine its potential for future clinical applications.

## RESULTS

### Catabolic protease expressionin human cartilage tissue

**Figure 1:**
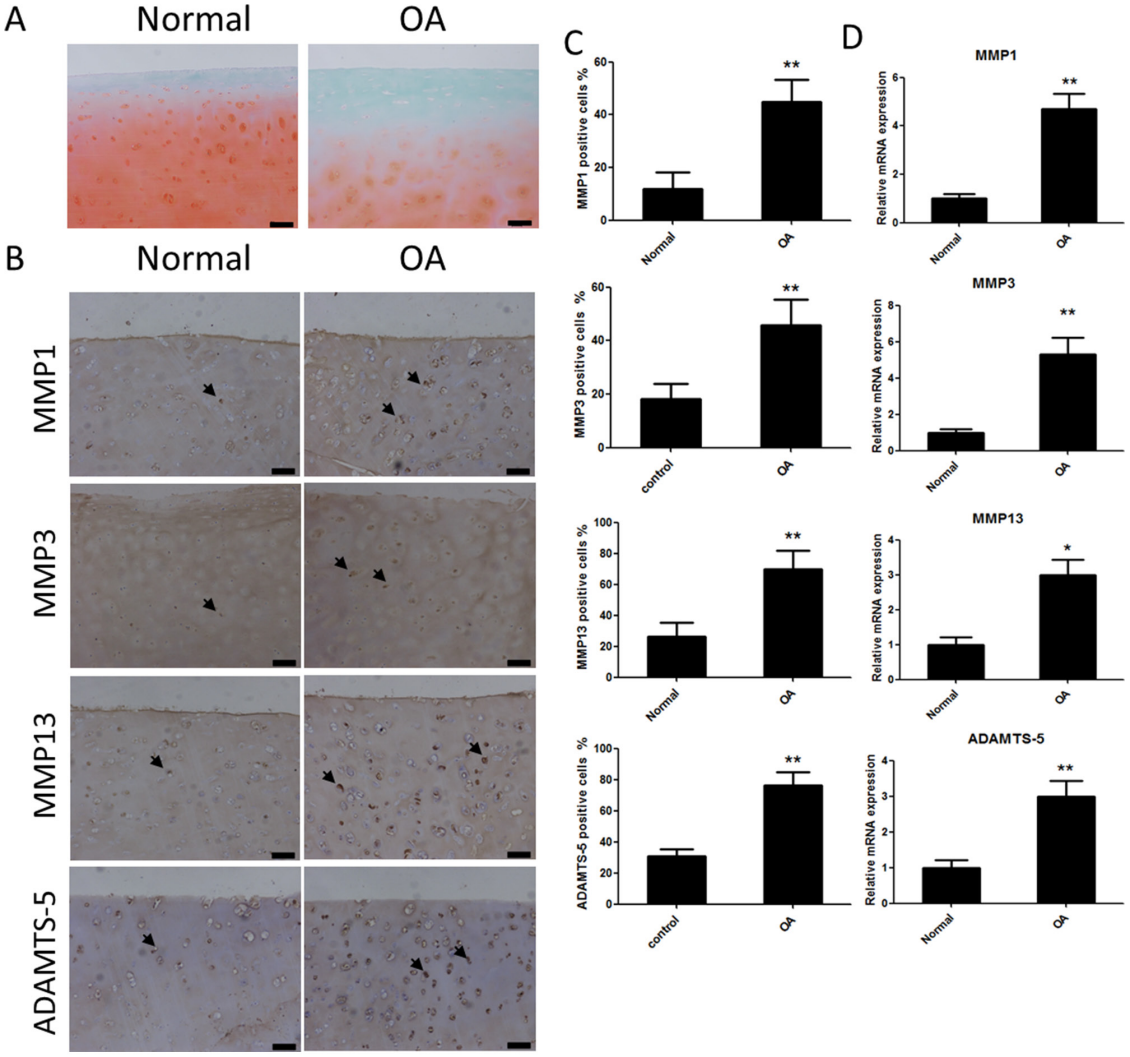
Safranin Orange staining and expression of catabolic proteases and extracellular matrix proteins in cartilage samples from human patients with or without osteoarthritis (OA) **(A)** Safranin Orange staining of cartilage samples from human patients with or without OA. Scale bar = 100 mm. **(B)** Immunohistochemistry of matrix metalloproteinase (MMP)-1, MMP3, MMP13 and A disintegrin and metalloproteinase with thrombospondin motifs (ADAMTS)-5 in normal and OA human cartilage samples. Scale bar = 100 mm. **(C)** Quantification of MMP1-, MMP3-, MMP13 and ADAMTS-5-positive cells in normal and OA human cartilage samples. **(D)** Relative mRNA expression of MMP1, MMP3, MMP13 and ADAMTS-5 in OA and normal human cartilage samples; n = 6, *p < 0.05 and **p < 0.01 (Student's *t*-test).

First, the expression of catabolic proteases in human cartilage tissue was evaluated. Safranin Orange staining was used to assess the cartilage samples. Compared with the cartilage samples from patients with OA, the cartilage tissue from healthy individuals contained more Safranin Orange-positive proteoglycan (Figure 1A). Second, the catabolic proteases were evaluated using immunohistochemistry. The results showed there were more catabolic proteases in the samples from patients with OA than in samples from patients without OA, based on the number of matrix metalloproteinase 1 (MMP1)-, MMP3-, MMP13-, and A disintegrin and metalloproteinase with thrombospondin motifs (ADAMTS)-5-positive cells (Figure 1B and 1C). The mRNA levels of MMP1, MMP3, MMP13, and ADAMTS-5 in the cartilage samples were consistent with the immunohistochemistry results (Figure 1D).

### Effects of SA on IL1-β-induced MMP1a, MMP3, MMP13, and ADAMTS-5 expression in chondrocytes

**Figure 2:**
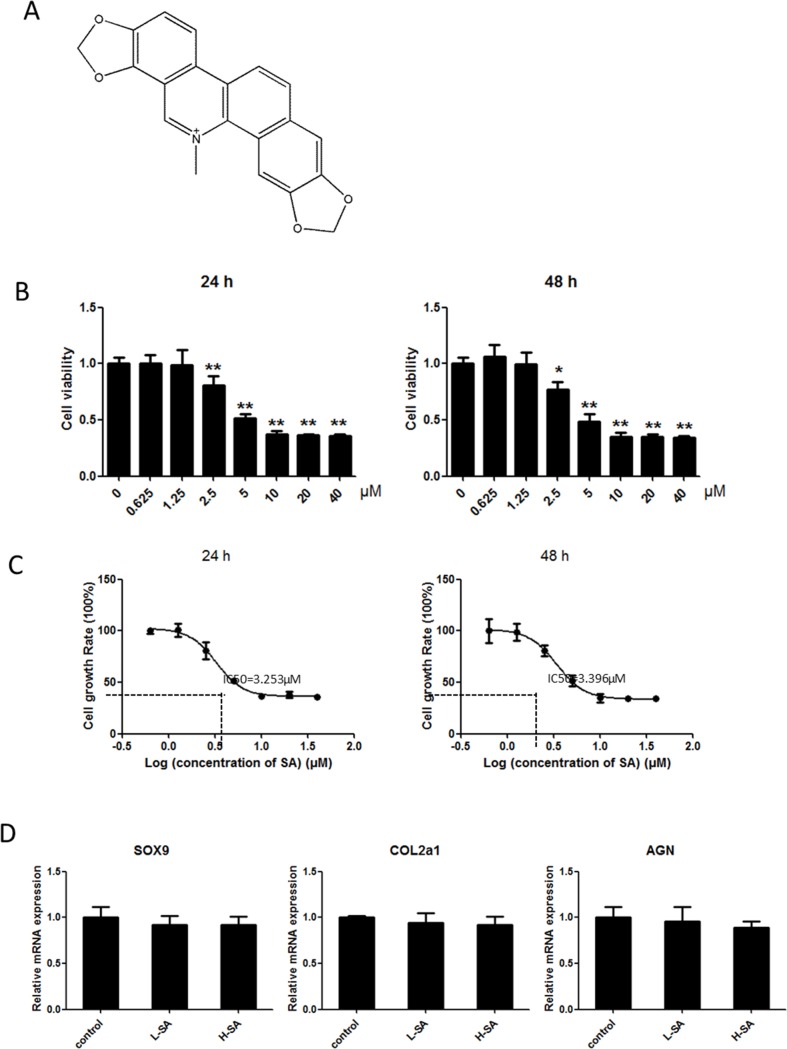
Effects of sanguinarine (SA) on viability of chondrocytes **(A)** Chemical structural formula of SA. **(B)** Cells were cultured with 0–40 μM SA for 24 or 48 h, and viability was determined using a cell counting kit (CCK)-8 assay. **(C)** Calculated half-maximal inhibitory concentration (IC_50_) values of SA in chondrocytes for 24 or 48 h was 3.253 and 3.396 μM, respectively. **(D)** Cells were cultured with 1.25 or 0.625 μM SA; subsequent expression of SRY-box 9 (SOX9), collagen type II α 1 (COL2A1), and aggrecan were detected using quantitative real-time polymerase chain reaction (qPCR); n = 6, *p < 0.05 and **p < 0.01 versus control (one-way analysis of variance, ANOVA).

**Figure 3:**
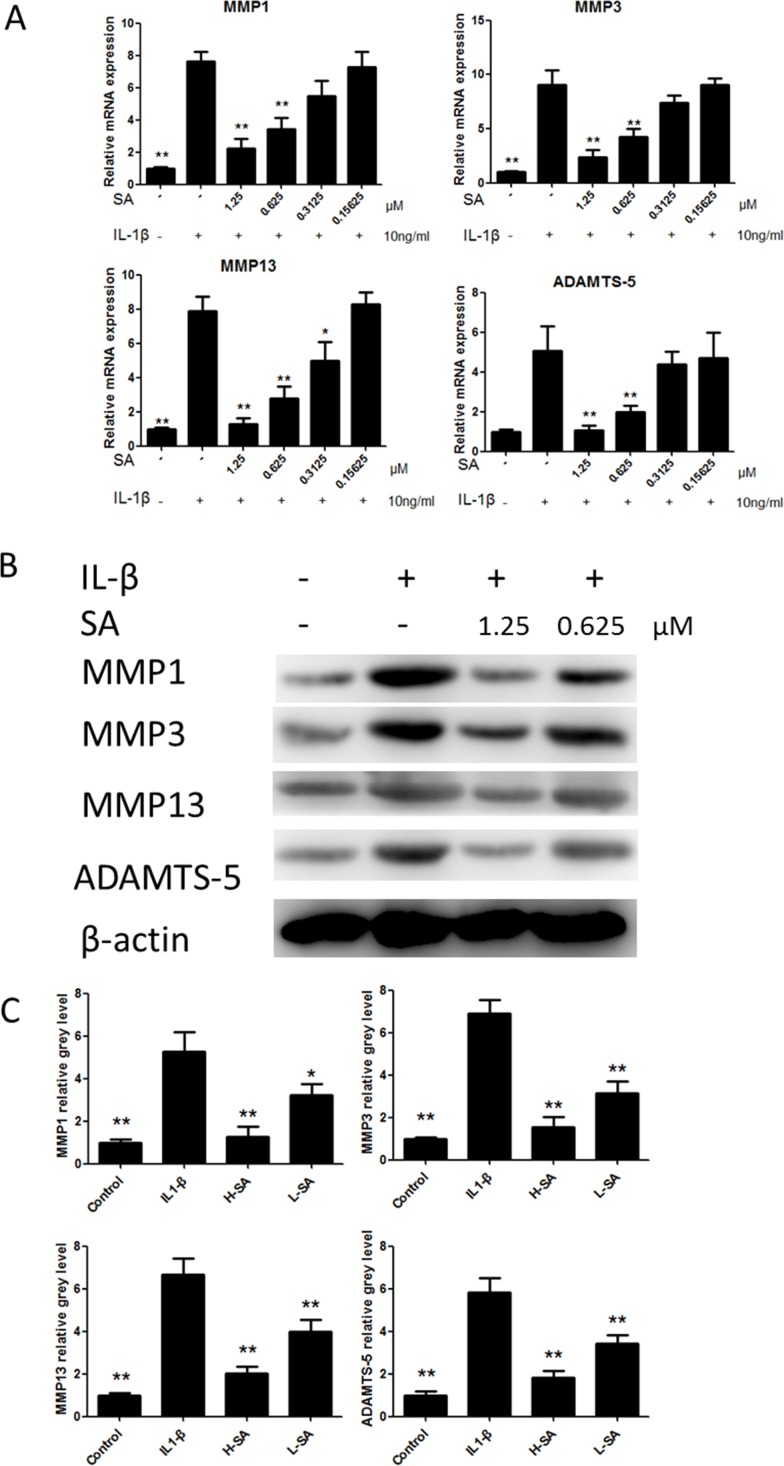
Sanguinarine (SA) inhibited interleukin (IL)-1β-induced matrix metalloproteinase-1 (MMP1), MMP3, MMP13, and A disintegrin and metalloproteinase with thrombospondin motifs (ADAMTS)-5 expression in chondrocytes **(A)** Effects of SA on mRNA expression of MMP1a, MMP3, MMP13, and ADAMTS-5 after treatment with IL-1β (10 ng/mL) for 24 h. **(B)** Western blot analysis of MMP1a, MMP3, MMP13, and ADAMTS-5 protein expression levels after treatment with different doses of SA (with or without IL-1 (10 ng/mL) stimulation) for 48 h. **(C)** Signal intensities of MMP1a, MMP3, MMP13, and ADAMTS-5 were detected using ImageJ program; n = 6, *p < 0.05 and **p < 0.01 versus IL-1β-stimulated group (one-way analysis of variance, ANOVA).

Before investigating the effects of SA on IL1-β-induced MMP1a, MMP3, MMP13, and ADAMTS-5 expression in chondrocytes, the potential cytotoxicity of SA against chondrocytes was evaluated using the cell counting kit-8 (CCK-8) assay. The results showed that cell viability was not affected by SA at concentrations lower than 1.25 μM at both 24 and 48 h (Figure 2B). The calculated half-maximal inhibitory concentration (IC_50_) values for SA in chondrocytes at 24 and 48 h were 3.253 and 3.396 μM, respectively (Figure 2C). Therefore, 1.25 and 0.625 μM were chosen as the high and low doses, respectively, for chondrocyte treatment. At these doses, SA had no effect on the expression of SRY-box 9 (Sox9), collagen type II α 1 (COL2A1), and aggrecan in chondrocytes (Figure 2D). As shown in Figure 3A, the mRNA expression of MMP1a, MMP3, MMP13, and ADAMTS-5 increased following stimulation with IL-1β (10 ng/mL). However, SA significantly decreased the expression of catabolic proteases in a dose-dependent manner. Similar changes were confirmed using western blot analysis, as shown in Figure 3B and 3C. Taken together, these results demonstrate that SA suppressed the expression of IL1-β-induced catabolic proteases in a dose-dependent manner.

### SA inhibits IL-1β-induced NF-κB and JNK activation in chondrocytes

**Figure 4:**
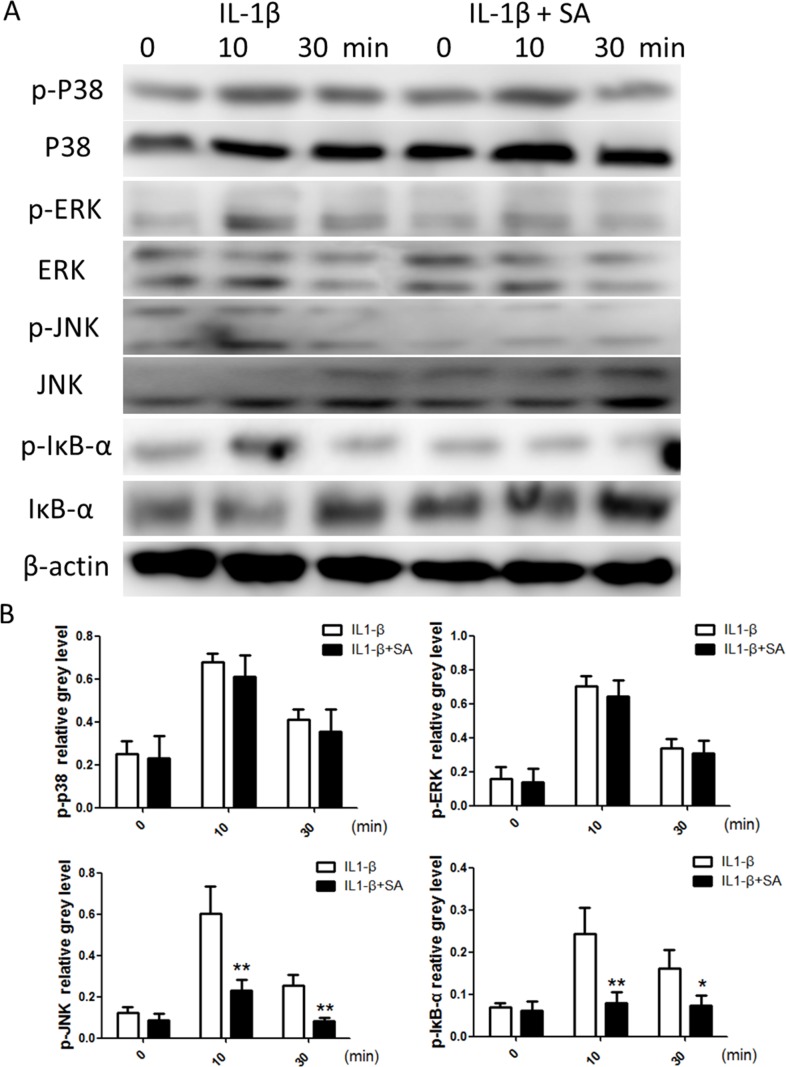
Sanguinarine (SA) inhibits interleukin (IL)-1β-induced nuclear factor (NF)-κB and c-Jun N-terminal kinase (JNK) activation in chondrocytes **(A)** Chondrocytes were treated with or without 1.25 μm SA for 4 h and then 10 ng/mL IL-1β for indicated time. Phosphorylated (p)-p38, extracellular signal-regulated kinase (ERK), JNK, inhibitor of NF-Κb (IκB)-α, total p38, ERK, JNK, IκB-α, and β-actin were evaluated using western blot analysis. **(B)** Signal intensities of p-P38, ERK, JNK, and IκB-α were quantified and normalized to total ERK, P38, JNK, and IκB-α using ImageJ, n = 6, *p < 0.05 and **p < 0.01 versus IL-1β-stimulated group (Student's *t*-test).

To elucidate the mechanism by which SA stimulated IL-1β-stimulated chondrocytes, we investigated the key signalling pathways such as those mediated by the mitogen-activated protein kinases (MAPKs), including extracellular signal-regulated kinase (ERK), p38, and Jun N-terminal kinase (JNK), and the NF-κB pathway, which are involved in the production of ADAMTSs and MMPs. As shown in Figure 4A, the MAPK and NF-κB pathways were clearly activated by IL-1β. However, phosphorylation of JNK and NF-κB decreased following treatment with SA. These observations were confirmed using quantitative analysis (Figure 4B). In contrast, SA showed no obvious effect on ERK and P38 activation. These results indicate that SA inhibited IL-1β-induced catabolic proteases, which involved the nuclear factor (NF)-κB and c-Jun N-terminal kinase (JNK) signalling pathways.

### Effect of SA on IL-1β-induced cartilage matrix degradation *ex vivo*

**Figure 5:**
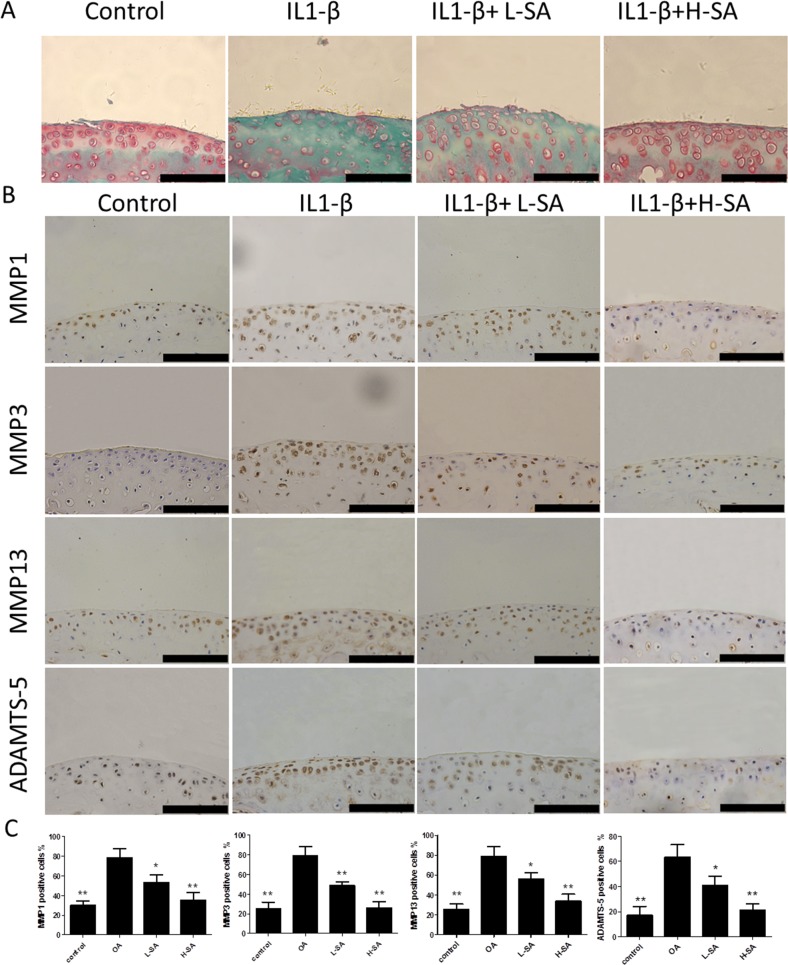
Effect of sanguinarine (SA) on interleukin (IL)-1β-induced cartilage matrix degradation *ex vivo* **(A)** Safranin Orange staining of *ex vivo* cartilage samples. Scale bar = 100 mm. **(B)** Immunohistochemistry was performed to assess expression of matrix metalloproteinase (MMP)-1a, MMP3, MMP13, and A disintegrin and metalloproteinase with thrombospondin motifs (ADAMTS)-5 in different samples. Scale bar = 100 mm. **(C)** Quantification of MMP1a-, MMP3-, MMP13-, and ADAMTS-5-positive cells in different samples. Scales bar = 100 mm; n = 6, *p < 0.05 and **p < 0.01 versus IL-1β-stimulated group (one-way ANOVA). Control, dimethyl sulphoxide (DMSO); IL-1β, 10 ng/mL; L-SA, 10 ng/mL IL-1β+ 0.625 μM SA; H-SA, 10 ng/mL IL-1β + 1.25 μM SA.

**Figure 6:**
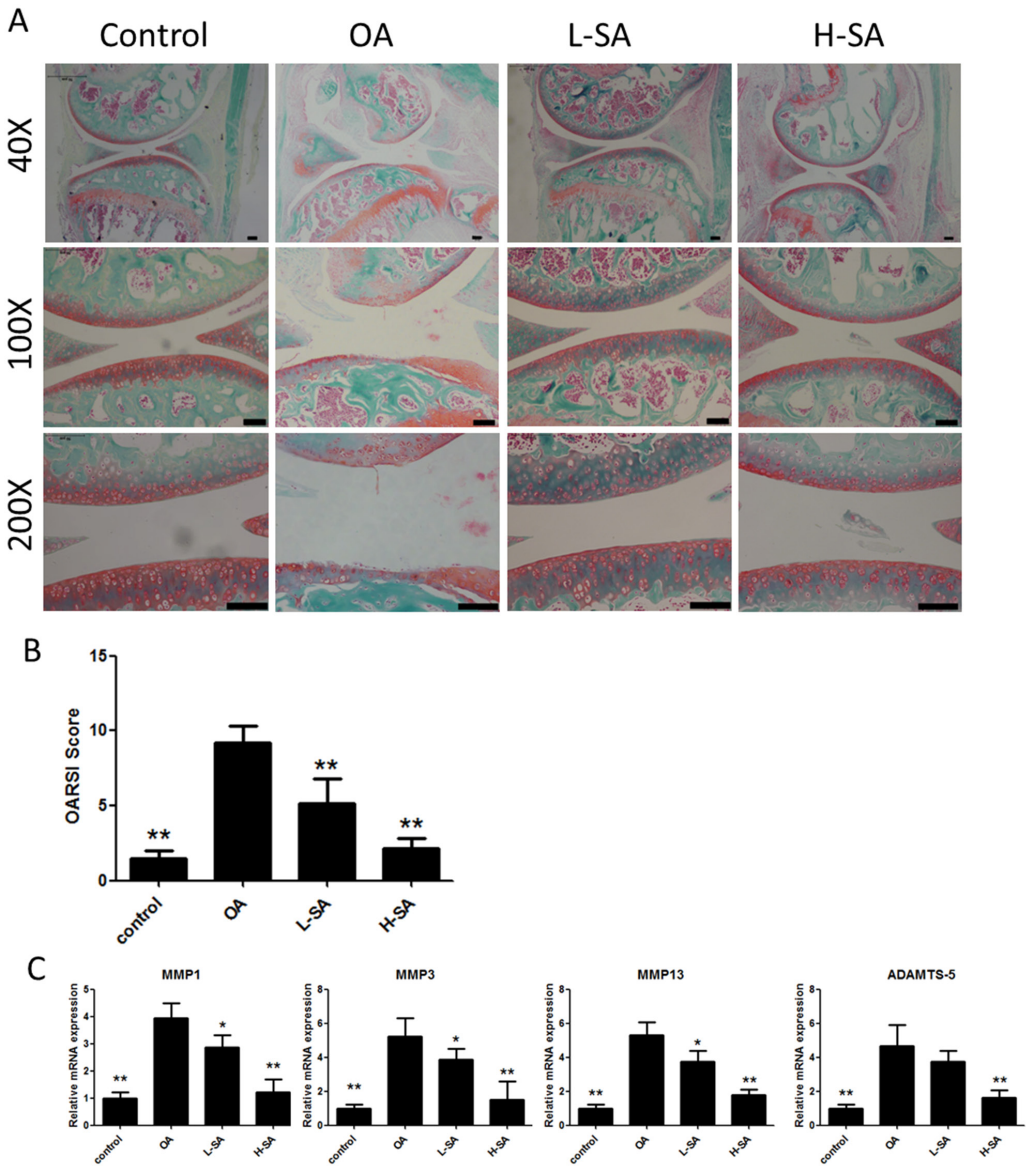
Effect of sanguinarine (SA) on anterior cruciate ligament transection (ACLT)-induced osteoarthritis (OA) **(A)** Safranin Orange staining of cartilage samples 6 weeks after OA induction. Scale bar = 100 mm. **(B)** OARSI score of samples 6 weeks after OA induction. **(C)** mRNA expression of matrix metalloproteinase (MMP)-1a, MMP3, MMP13 and A disintegrin and metalloproteinase with thrombospondin motifs (ADAMTS)-5 in different groups; n = 6, *p < 0.05 and **p < 0.01 versus OA group (one-way analysis of variance, ANOVA). Control, sham group; OA, ACLT-induced OA group; L-SA, ACLT-induced OA treated with 0.625 μM intra-articularly administered SA twice a week; H-SA, ACLT-induced OA treated with 1.25 μM intra-articularly administered SA twice a week.

Considering that SA inhibited the IL-1β-induced catabolic protease expression *in vitro*, we hypothesized that it could prevent IL-1β-induced cartilage matrix degradation *ex vivo*. Therefore, mouse knee joint cartilage was placed in an explant culture medium and stimulated with IL-1β to induce matrix degradation. Samples were then randomly divided into four groups and treated with PBS, IL-1β, and IL-1β with low or high concentration SA. The level of matrix degradation was evaluated using Safranin Orange staining. Compared to the IL-1β-treated group, the cartilage of the group simultaneously treated with IL1-β and SA exhibited more Safranin Orange-positive proteoglycan, which was dose-dependent (Figure 5A). We further investigated the expression of catabolic proteases in cartilage matrix using immunohistochemistry. Quantitative analysis demonstrated that SA significantly decreased the number of MMP1a-, MMP3-, MMP13-, and ADAMTS-5 positive cells (Figures 5A and 6B). The *ex vivo* findings indicate that SA protected cartilage against degradation partly by inhibiting MMP1a, MMP3, MMP13, and ADAMTS-5.

### Effect of SA on anterior cruciate ligament transection (ACLT)-induced OA

**Figure 7:**
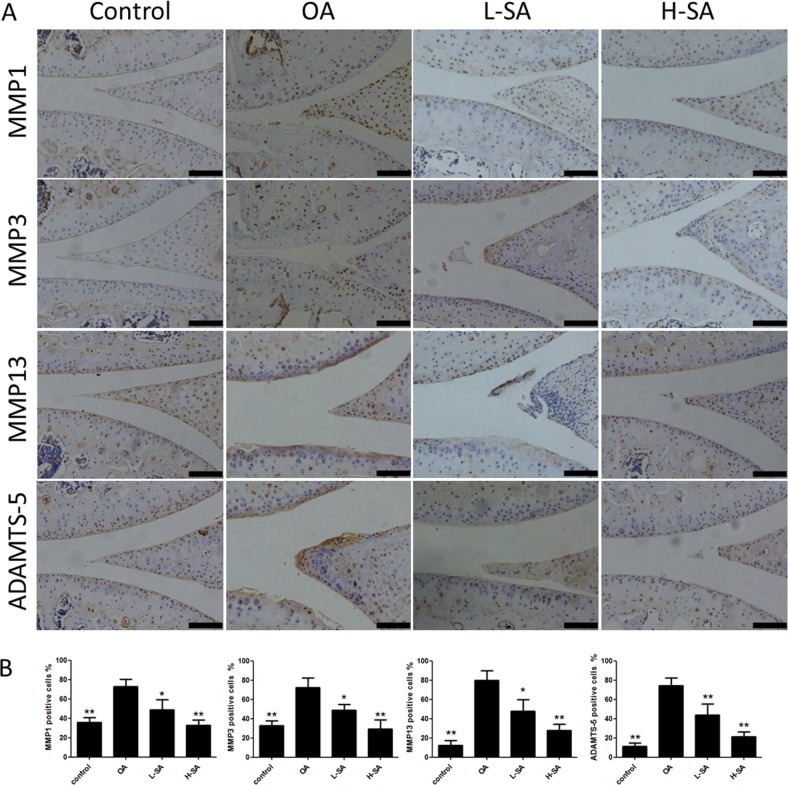
Effect of sanguinarine (SA) on cartilage matrix degradation *in vivo* **(A)** Immunohistochemistry of matrix metalloproteinase (MMP)-1a, MMP3, MMP13, and A disintegrin and metalloproteinase with thrombospondin motifs (ADAMTS)-5 in cartilage samples 6 weeks after surgery. Scale bar = 100 mm. **(B)** Quantification of MMP1a-, MMP3-, MMP13-, and ADAMTS-5-positive-cells in cartilage samples 6 weeks after surgery; n = 6, *p < 0.05 and **p < 0.01 versus osteoarthritis (OA) group (one-way analysis of variance, ANOVA).

The preceding results demonstrated the effect of SA on chondrocytes *in vitro* and *ex vivo*. To determine whether SA delayed the progression of OA *in vivo*, low and high concentrations were administered intra-articularly to mice with anterior cruciate ligament transection (ACLT)-induced OA. Six weeks after surgery, Safranin Orange staining was used to evaluate the degradation of the cartilage matrix. SA improved the cartilage surfaces of ACLT-induced OA mice in a dose-dependent manner (Figure 6A). Quantitative analysis using the Osteoarthritis Research Society International (OARSI) scoring indicated that even a low concentration of SA (0.625 μM) yielded significantly lower OARSI scores than those reported for the OA group (Figure 6B). The mRNA expression levels of MMP1a, MMP3, MMP13, and ADAMTS-5 were evaluated in each group (Figure 6C). The OA group showed higher levels of MMP1a, MMP3, MMP13, and ADAMTS-5 expression than the sham surgery control group did. Furthermore, treatment with SA decreases all of the catabolic proteases investigated and the immunohistochemistry results were consistent with the mRNA data (Figure 7A and 7B). These results indicated that SA could inhibit OA progression *in vivo*.

## DISCUSSION

SA has a long history of use in the treatment of infections and as an analgesic, expectorant, sedative, and emetic [20]. Recently, the anti-inflammatory effects of SA were reported in animal models of acetic acid-induced colitis and lipopolysaccharide (LPS)-induced endotoxic shock [21, 22]. The present study showed, for the first time, that SA might be useful in treating OA.

Inflammatory cytokines are involved in the pathogenesis of OA. IL-1β is generated by inflamed synovium and invading leukocytes, and induces the release of MMPs and ADAMTS from both the synovium and cartilage [5, 23, 24]. This phenomenon is supported by our results, which showed that the expression of MMP1, MMP3, MMP13, and ADAMTS-5 was higher in samples from patients with OA than in those from patients without OA (Figure 1A). ADAMTS and MMPs are catabolic proteases capable of degrading all types of extracellular matrix proteins, and the proteoglycan aggrecan and COL2 are the two major structural components of the cartilage extracellular matrix [25]. In particular, ADAMTS-5 is regarded as the main enzyme contributing to the degradation of aggrecan [26, 27]. Among the MMPs, MMP1, MMP3, and MMP13 are the main proteases involved in the degradation of COL2 [28, 29]. Our results showed that SA decreased the expression of MMP1, MMP3, MMP13, and ADAMTS-5 in chondrocytes.

ERK, JNK, and the NF-κB signalling pathways are well-known to be involved in the pathogenesis of OA [30, 31]. NF-κB and JNK signalling are the major upstream mediators of the expression of catabolic factors [32]. It has been shown that aggrecan degradation is mainly regulated by the JNK2 pathway [33]. Previous studies have demonstrated that the inhibition of MAPK and NF-κB activity reduces the production of inflammatory mediators in chondrocytes [8, 10]. In concurrence with these studies, we found that SA suppressed IL1-β-induced NF-κB and JNK activation, which presented a high level of specificity in repressing the production of catabolic factors. This finding is partially supported by the results of Chaturvedi et al. [34] that SA is a potent suppressor of NF-κB activation In addition, Niu et al. [35] found that SA inhibited the phosphorylation of p38 and ERK, which altered the synthesis and release of inflammatory mediators in peritoneal macrophages. In contrast, SA had no influence on p38 and ERK in our study. This is because the study by Niu et al. [35] investigated the effects of SA using peritoneal macrophages, while we demonstrated the SA-induced inhibition of IL1-β-induced NF-κB and JNK activation in chondrocytes.

There is considerable scope for further investigation of the effects of SA against OA. First, we investigated the effects of SA on chondrocytes alone. The remodelling of subchondral bone and the associated inflammation in the synovium are typical pathological features of OA, and recent studies have shown that SA inhibited the formation of osteoclasts. Therefore, an investigation focusing on the subchondral bone and synovium would be required to completely elucidate the role of SA in OA. Furthermore, decreasing the frequency of intra-articular treatment administration with a more practical delivery system will be valuable for potential clinical applications. In conclusion, our study showed that SA suppressed the expression of catabolic proteases *in vitro*, *ex vivo*, and *in vivo*, indicating its potential value in the treatment of OA.

## MATERIALS AND METHODS

### Chemicals and reagents

SA and dimethyl sulphoxide (DMSO) were purchased from Sigma-Aldrich (St Louis, MO, USA). Dulbecco's modified Eagle's medium (DMEM), foetal bovine serum (FBS), and penicillin/streptomycin were purchased from Gibco-BRL (Gaithersburg, MD, USA). The CCK-8 was obtained from Dojindo Molecular Technology (Kumamoto, Japan). SA was dissolved in DMSO and stored at −20°C. The experiments were carried out in the absence of visible light to prevent photosensitivity. SA was diluted with cell culture medium to obtain a final DMSO concentration < 0.1% of the total volume. The following antibodies against ERK (sc-292838), c-Jun N-terminal kinase (JNK, sc-572), p38 (sc-535), phospho-ERK (Thr202/Tyr204, sc-16981), phospho-JNK (Thr183/Tyr185, sc-6254), phospho-p38 (Thr180/Tyr182, sc-17852), MMP3 (sc6839), MMP-13 (sc30073), ADAMTS-5 (sc83186), aggrecan (sc25674), and β-actin (sc25674) were from Santa Cruz (Santa Cruz, CA, USA). While antibodies against IκB-α (ab32518), phospho-IκB-α (ab133462), MMP1 (ab137332), and Col2A1 (ab34712) were purchased from Abcam (Cambridge, UK). All other reagents were purchased from Sigma-Aldrich (St Louis, MO, USA) unless stated otherwise.

### Human cartilage

Human cartilage samples were collected according to protocols approved by the Ethics Committee of Sir Run Run Shaw Hospital and the methods were carried out in accordance with the approved guidelines. Written informed consent was obtained from all subjects. Six control (healthy) cartilages were harvested from human cadavers without a history of OA while six pathological cartilages were acquired from patients with end-stage symptomatic knee OA at the time of surgery for total knee replacement. The human cartilage samples were graded based on the modified 5-point Collins scale. In the OA group, all the samples were classified as grade 3 while those in the control group were all grade 0. Human cartilage samples were obtained using previously described methods [13].

### Cell culture

Primary articular cartilage was isolated from the femoral condyles and tibial plateaus of C57B/6 mice on postnatal day 5–6, according to a previous study [36]. Chondrocytes were cultured in DMEM containing 10% (v/v) FBS, 100 U/mL penicillin, and 100 μg/mL streptomycin at 37°C with 5% CO_2_. Third-passage cartilage cells were used in all of the experiments.

### Cell viability assay

The cytotoxic effects of SA were assessed using CCK-8 assays. Chondrocytes were seeded in 96-well plates at a density of 8 × 10^3^ cells/well in triplicate and cultured in complete DMEM for 24 h. The cells were treated with different concentrations of SA (0, 0.15625, 0.3125, 0.625, 1.25, 2.5, 5, 10, 20, or 40 μM) for 24 or 48 h, 10 μL CCK-8 buffer was added to each well, and then the cells were incubated at 37°C for 2 h. This was followed by measurement of the absorbance at 450 nm (650 nm reference) using an ELX800 absorbance reader (Bio-Tek, Winooski, VT, USA). The cell viability was calculated relative to that of the control cells from the optical density (OD) data using the following formula: cell viability = (experimental group OD – zeroing OD)/(control group OD – zeroing OD). The experiments were performed six times, and the significance was determined as indicated in the figure legend.

### Quantitative real-time polymerase chain reaction (qPCR)

Total RNA was extracted from chondrocytes or cartilage samples using the TRIzol reagent (Invitrogen Inc., Carlsbad, CA, USA) according to the manufacturer's instructions. cDNA was synthesized from 1 μg total RNA using reverse transcriptase (TaKaRa Biotechnology, Otsu, Japan). The real-time polymerase chain reaction (PCR) was performed using the SYBR Premix Ex Taq kit (TaKaRa, Biotechnology, Otsu, Japan) and an ABI 7500 sequencing detection system (Applied Biosystems, Foster City, CA, USA). The following cycling conditions were used: 40 cycles of denaturation at 95°C for 5 s and amplification at 60°C for 24 s. The relative level of expression of each target gene was calculated using the 2^−ΔΔCT^ method. Each real-time PCR included six different experimental samples. The data were represented as target gene expression normalized to the reference gene. Error bars indicate the SD of the mean of replicates. All pairs of primer sequences were designed using the primer 5.0 software and are listed in Table 1.

**Table 1:** Polymerase chain reaction (PCR) primers

Gene	Strand	Primer sequences	Origin
*GAPDH*	forward	5′-AGGGCCCTGACAACTCTTTT-3′	human
	reverse	5′-AGGGGTCTACATGGCAACTG-3′	
*MMP1*	forward	5′-CAAGTGCTGTGGCACTGTTT-3′	human
	reverse	5′-ACTTGTCCCAGCATTGAACC-3′	
*MMP3*	forward	5′-CTGGGAAAATCAGCCATTGT-3′	human
	reverse	5′-AGGTTCTGGAGGGACAGGTT-3′	
*MMP13*	forward	5′-AACATCCAAAAACGCCAGAC-3′	human
	reverse	5′-TTGGCAATATGCAGAAGCAG-3′	
*ADAMTS-5*	forward	5′-TAGGCGTCTTTGGCTTCAGT-3′	human
	reverse	5′-GCTTTCAGGCTGACCAGTTC-3′	
*Col2A1*	forward	5′-CAACCAGACCGTGCTTTTCA-3′	human
	Reverse	5′-CAACCGTAGCAATCGACCAG-3′	
*Aggrecan*	forward	5′-TCCCCGTAGAAGAGGAGACA-3′	human
	reverse	5′-AAGACAGGGGTATGCAGCTT-3′	
*GAPDH*	forward	5′-ACCCAGAAGACTGTGGATGG-3′	mouse
	reverse	5′-CACATTGGGGGTAGGAACAC-3′	
*MMP1a*	forward	5′-CTAAGGCAGGAGGATTGCTG-3′	mouse
	reverse	5′-TGCGAAGGGCTTAGTGTCTT-3′	
*MMP3*	forward	5′-CTCAGAGGAGCAAGGGTTTG-3′	mouse
	reverse	5′-CAACTGCGAAGATCCACTGA-3′	
*MMP13*	forward	5′-GAGCCACAGATGAGCACAGA-3′	mouse
	reverse	5′-ATGTAAGGCCACCTCCACTG-3′	
*ADAMTS-5*	forward	5′-CAAGTTAGCTGGCTGGGAAG-3′	mouse
	reverse	5′-TCCAGATGCAGCAAACAAAG-3′	
*Sox 9*	forward	5′-AGAAAGGAGTGAGGGGGTGT-3′	mouse
	reverse	5′-TGCCGGTTCTCAGTAGCTTT-3′	
*Col2A1*	forward	5′-CTGGGCCACAGGTGAGTATT-3′	mouse
	reverse	5′-CCAGGATACCAACTGCCTGT-3′	
*Aggrecan*	forward	5′-AAGGACGAGTTCCCTGGAGT-3′	mouse
	reverse	5′-CTGGGGATGTCGCATAAAAG-3′	

### Western blot analysis

To investigate the effect of SA on the IL1-β-induced expression of MMP1, MMP3, MMP13, and ADAMTS-5, chondrocytes were seeded in six-well plates at a density of 5 × 10^5^ cells/well. When the cells were confluent, they were treated with SA with and without IL-1 (10 ng/mL) stimulation, for 48 h. Then, total protein was extracted from cultured cells using radio-immunoprecipitation assay (RIPA) lysis buffer (Sigma-Aldrich, St Louis, MO, USA) containing 50 mM Tris-hydrochloride (HCl), 150 mM sodium chloride (NaCl), 5 mM ethylenediaminetetraacetic acid (EDTA), 1% Triton X-100, 1 mM sodium fluoride, 1 mM sodium vanadate, 1% deoxycholate, and a protease inhibitor cocktail. Lysates were centrifuged at 12,000 × g for 15 min, and the supernatants that contained the proteins were collected.

To examine the target signalling pathway of SA, chondrocytes were seeded in six-well plates at a density of 5 × 10^5^ cells/well. When the cells were confluent, they were either untreated or pre-treated with 1.25 μM SA for 4 h. The cells were then stimulated with 10 ng/mL IL1-β for 0, 10, and 30 min, followed by the collection of the total protein as above.

Protein concentrations were determined using the bicinchoninic acid (BCA, Thermo Fisher, Waltham, MA, USA) assay. Then, 30 μg of each protein lysate was resolved using 10% sodium dodecyl sulphate-polyacrylamide gel electrophoresis (SDS-PAGE, Sigma-Aldrich, St Louis, MO, USA) and then transferred to polyvinylidene difluoride membranes (Millipore, Bedford, MA, USA). Then, the membranes were blocked with 5% skimmed milk in Tris-buffered saline plus Tween (TBST, 0.05 M Tris/0.15 M NaCl [pH 7.5] and 0.2% Tween-20; Invitrogen, San Diego, CA, USA) for 1 h, and then incubated with primary antibodies diluted in 1% (w/v) skimmed milk in TBST overnight at 4°C. The membranes were then washed and incubated with the appropriate secondary antibodies conjugated with IRDye 800CW (molecular weight: 1162 Da). The antibody reactivity was detected via exposure in an Odyssey infrared imaging system (LI-COR, Inc., Lincoln, NE, USA). Quantitative analysis of the band intensity was carried out using the ImageJ software.

### Cartilage explant cultures

The animal experiments in this study were performed in accordance with the principles and procedures of the National Institutes of Health (NIH) Guide for the Care and Use of Laboratory Animals and the guidelines for animal treatment of Sir Run Run Shaw Hospital. All experimental protocols in this study were approved by the Ethics Committee of Sir Run Run Shaw Hospital. Femoral condyle cartilage was obtained from 12-week-old C57BL/6 mice without the bone, periosteum, or synovium and cultured in DMEM containing 10% FBS (v/v), antibiotics, and 2 mM L-glutamine (Invitrogen) in 5% CO_2_ at 37°C. After 2 days of culturing, the explants were washed with serum-free medium and dispensed into tissue-culture dishes with serum-free medium containing 10 ng/mL IL1-β with or without 0.625 or 1.25 μM SA. Furthermore, there were six explants in each group, and after a 72-h incubation, the samples were removed from the culture wells and fixed in 10% formalin.

### ACLT surgery-induced OA and intra-articular delivery of SA

Twenty-four 8-week-old male C57BL/6 mice were divided into four groups and those in group I underwent a sham knee surgery. The mice in the other three groups were subjected to ACLT surgery of the knees, as previously described [37]. Then, 10 days later, intra-articular injections of normal saline (group II), low- and high-dose SA (0.625 and 1.25 μM, groups III and IV, respectively) were administered to the mice twice a week. Six weeks later, the distal femur of each mouse was dissected, embedded in paraffin, and examined using Safranin Orange and immunostaining.

### Histological analysis and immunohistochemistry of murine knee joints

The knee joints were examine using histological and immunohistochemical assays. We used 4% (v/v) neutral buffered formalin to fix the tissue samples for 24 h and neutral 10% EDTA solution to decalcify for 1 month at room temperature. Next, the samples were dehydrated in a graded series of ethanol solutions, embedded in paraffin blocks, and sagittal serial sections were cut from across the entire knee joint. Six 8-μm histological sections from different depths of each joint were cut at five-slide intervals, stained with Safranin O-fast green, and then the OARSI histopathology grading and staging system was used to grade the tissue degeneration [38]. Five sequential sections per joint from each group were immunostained. The sections were incubated at 4°C with antibodies against MMP1, MMP3, MMP13, and ADAMTS-5 overnight, and then incubated for 2 h at room temperature with the secondary antibodies (Beyotime Institute of Biotechnology Inc., Jiangsu, China). The number of positively stained cells on the entire articular surface (including the femoral condyle and tibial plateau area) per specimen was counted and the percentage positive cells was calculated [12].

### Statistical analysis

All data are presented as the mean ± standard deviation (SD). The statistical data were analysed using a one-way analysis of variance (ANOVA)/Tukey's post-hoc test or Student's *t*-tests. A *p < 0.05 or **p < 0.01 was considered statistically significant.
